# Diversity of *Mycobacterium tuberculosis* Complex Lineages Associated with Pulmonary Tuberculosis in Southwestern, Uganda

**DOI:** 10.1155/2021/5588339

**Published:** 2021-06-25

**Authors:** Lisa Nkatha Micheni, Kennedy Kassaza, Hellen Kinyi, Ibrahim Ntulume, Joel Bazira

**Affiliations:** ^1^Department of Microbiology, Mbarara University of Science and Technology, P.O. Box 1410 Mbarara, Uganda; ^2^Department of Microbiology and Immunology, Kampala International University Western Campus, P.O. Box 71, Bushenyi, Uganda; ^3^Department of Biochemistry, School of Medicine, Kabale University, P.O. Box 317, Kabale, Uganda

## Abstract

Uganda is among the 22 countries in the world with a high burden of tuberculosis. The southwestern region of the country has consistently registered a high TB/HIV incidence rate. This study is aimed at characterizing the *Mycobacterium tuberculosis* complex (MTBC) genotypic diversity in southwestern Uganda. A total of 283 sputum samples from patients with pulmonary tuberculosis were genotyped using specific single nucleotide polymorphism markers for lineages 3 and 4. Most of the patients were males with a mean age of 34. The lineage 4 Ugandan family was found to be the most dominant strains accounting for 59.7% of all cases followed by lineage 3 at 15.2%. The lineage 4 non-Ugandan family accounted for 14.5% of all cases while 4.2% showed amplification for both lineage 4 and lineage 3. Eighteen samples (6.4%) of the strains remained unclassified since they could not be matched to any lineage based on the genotyping technique used. This study demonstrates that a wide diversity of strains is causing pulmonary tuberculosis in this region with those belonging to the lineage 4 Ugandan family being more predominant. However, to confirm this, further studies using more discriminative genotyping methods are necessary.

## 1. Introduction

Tuberculosis (TB) is an ancient communicable disease that has been in existence over the past millennia and up to date remains a major global public health problem. It is caused by closely related acid-fast bacteria known as *Mycobacterium tuberculosis* complex (MTBC) with seven human-adopted MTBC lineages (designated lineage 1 through lineage 7) and two lineages adapted to various wild and domestic animal species but capable of causing human infection being implicated [1–4]. Epidemiological studies reveal that there is a strong phylogeographical structuring of this organism [5–7] and well adapted to sympatric human populations [8, 9]. This makes these lineages more predominant in specific human populations of certain geographical locations [1, 7, 10]. For instance, lineage 2 (L2) is most predominant in East Asia and is more associated with virulence and drug-resistant than other lineages [11, 12]. L1 (moderately virulent) and L3 mainly occur in areas around the Indian Ocean whereas L5 and L6 are highly restricted to West Africa [13, 14]. L4 is commonly found in Africa, Central America, Europe, and South America [15], while L7 is highly and exclusively restricted in Ethiopia [16]. This adoption could be a result of coevolution [17, 18]. Studies have also shown that human migration greatly contributes to the spread of TB and causes an increase in the genetic diversity of MTBC [9, 19–23].

Uganda is listed among the world's 22 countries with the highest TB burden countries [24] and the third-largest refugee host country in the world after Turkey and Pakistan with over 1.36 million refugees [25–27]. The refugees come from countries such as the Democratic Republic of the Congo (DRC), Burundi, Ethiopia, Eritrea, Rwanda, Somalia, and South Sudan. Over the past five years, there has been a mass refugee influx from DRC and South Sudan into the country with the southwestern region of the country accommodating most of these refugees. Ninety-two percent (92%) of these refugees live alongside the local communities where accommodation and farming land are given to them [27]. Earlier studies conducted in central Uganda indicate that majority of the TB cases are due to MTBC lineage 4 sublineage Ugandan family (L4-U) [28–30], Euro-American lineage. This sublineage is defined by a deletion in 724 region of difference (RD) and (SNP) typing 33–36, 40 and 43 spoligotype fingerprint spacers missing, and several single nucleotide polymorphisms (SNPs). Similarly, a 2010 study in southwestern Uganda done by Bazira and colleagues using spoligotyping indicated that the majority of TB strains in the region belong to the Uganda genotype [31]. However, the fact that there has been an influx of immigrants in a region that consistently registers high TB incidence rates calls for a greater understanding of the molecular epidemiology and genetic variability of MTBC in the region to enable better control of this causative agent of TB.

## 2. Methods

### 2.1. Study Setting

This study was conducted between May 2018 and April 2019 in the southwestern region of Uganda, a region with fifteen administrative districts and borders Tanzania, Rwanda, and the Democratic Republic of Congo (Supplementary File 1: Figure 1). The region is heavily affected by the TB/HIV epidemic and constantly registers high TB incidence rates [32–34]. There were four patient recruitment centers which included two regional referral hospitals (Kabale and Mbarara regional referral hospitals) and two health centers within the refugee camps in the region (Oruchinga and Nakivale health center IV).

### 2.2. Patient Recruitment and Sample Collection

The patient's clinical information including age, sex, HIV status, previous history of TB, and economic status was recorded. Sputum samples were collected from patients aged ≥18 years. Cases of pulmonary tuberculosis (PTB) were diagnosed at the sample collection centers by either GeneXpert Cepheid test for samples analyzed at the regional referral hospitals or sputum smear microscopy for samples analyzed in the health centers. Those samples diagnosed positive for PTB were then transported (not more than 72 hours after collection time) in a cold box to Mbarara University of Science and Technology Genomics and Translational Laboratory for processing and molecular analysis.

### 2.3. DNA Extraction and Confirmation of MTB in Sputum Samples

The genomic DNA from each patient sample was processed by standardized protocols [35, 36]. All the samples were then screened and confirmed as MTB by detection of a 123 bp fragment of the IS*6110* gene which is common among the members of the MTBC.

### 2.4. Single Nucleotide Polymorphism (SNP) Typing

SNP typing to determine the MTB lineages was performed by RT-PCR (Bio-Rad CFX96 Touch™). Lineage-specific primers were as follows: Rv004C for MTB L4-U, Rv2962C for MTB L4-NU, and Rv0129C for MTB L3 based on a previous study of Wampande et al. [37] and their accompanying hybridization probes (Supplementary File 2: Table 1). The MTBC lineages were identified based on differences in melting temperature (*T*_m_). Briefly, the assays were performed in 20 *μ*l reaction mixture containing 3.75 *μ*l of PCR water, 1.25 *μ*l (0.5 *μ*M final concentration) of each primer, 0.625 *μ*l (0.25 *μ*M final concentration) of each probe, 9.5 *μ*l of 2X Lunar® Universal genotyping master mix, and 3 *μ*l (5–50 ng) of extracted genomic DNA. The Bio-Rad CFX96 Touch™ Real*-*Time PCR Detection System was programmed for PCR amplification and a melting curve stage. For each of the three uniplex assays, the amplification stage consisted of a pre-PCR stage performed at 95°C for 10 min, an amplification stage with denaturation at 95°C for 30 s, primer annealing (50°C for Rv004C or 52°C for Rv0129C or 51°C for Rv2962C) for 30 s, and extension at 60°C for 30 s for 45 cycles. The melting curve analysis consisted of denaturation of the amplicons at 95°C for 10 s to produce single-stranded DNA, probe annealing temperature at 65°C for 05 s with a continuous acquisition mode to allow capture of the fluorescence, and probe melting temperature ranging from 40 to 80°C. In all the assays, kc32969 (L4-U), H37Rv (L4-NU), and delicus (L3) genomic DNA (courtesy of Makerere Molecular Labs) were used as positive control while nontemplate mix as a negative control. The Bio-Rad CFX96 Touch™ software was used to determine the MTB lineages through the analysis of amplicon melting temperature (*T*_m_) (Supplementary File 3: Figure 2).

### 2.5. Statistical Analysis

Patient demographic data were converted into Excel tables and then exported to SPSS version 25 (IBM, Chicago, USA) for analysis. Bivariate analysis was performed using the chi-square test to determine the relationship between categorical variables (independent variables) and dependent variables. Multinomial logistic regression models were fitted to evaluate the relationship between MTB lineage (dependent variable) and patients' country of origin/ethnicity (primary independent variable). Patients' characteristics of age, gender, and economic status were treated as covariates when fitting the final model. Statistical significance was considered at *p* ≤ 0.05. Chi-square and Fisher's exact tests were computed, and a *p* value of ≤0.05 was considered evidence of a significant difference.

## 3. Results

**Figure 1:**
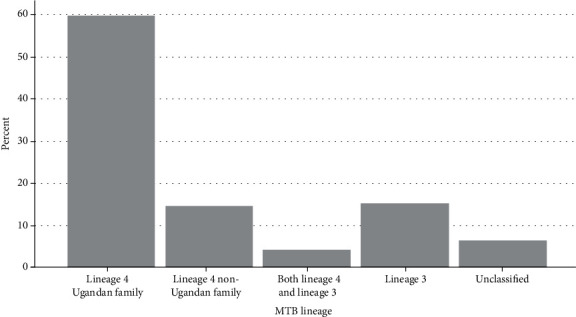
Overall prevalence of MTBC lineages causing PTB in southwestern Uganda.

A total of 283 samples were genotyped. Majority of the samples (246, 86.9%) were obtained from Ugandan patients. The mean age of the patients was 34 years, and most (73.1%) were males. One hundred and twenty-nine (45.6%) of the patients had unknown serostatus at the time of the study and did not consent to test while 26.9% were HIV seropositive. Among the 283 samples analyzed, 59.7% (169) belonged to lineage 4 Ugandan family (L4-U), 14.5% (41) were other lineage 4 non-Ugandan (L4-NU), and 15.2% (43) lineage 3 (L3) while 4.2% (12) had amplification for both lineage 4 and lineage 3. The remaining 18 (6.4%) strains could not be matched to any lineage based on the genotyping techniques used (Figure 1). Of the 37 samples from the refugees, 8.1% belonged to L4-U family while 1.1%, 3.2%, and 0.4% were L4-NU, L3, and unclassified strains, respectively. In those samples from the Ugandan patients, 51.6% belonged to L4-U family while 13.4%, 12%, and 6% were L4-NU, L3, and unclassified strains, respectively (Table 1).

Bivariate analysis taking lineage as the outcome showed that the proportions of patients infected with each MTBC lineage did not differ according to age, sex, HIV status, level of income, or history of TB in the past. Despite the fact that the Ugandan genotype was found in a higher proportion of those confirmed by GeneXpert or microscopy, the proportion was not statistically significant (*p* = 0.074) (Table 1). A multinomial logistic regression analysis was further performed to determine the relationship between the independent variable “patients' nationality” and the MTB lineages circulating in southwestern Uganda (Table 2). It was revealed that the Ugandan patients were significantly likely to have L4-U strains (OR: 0.501; 95% CI: 0.143-1.758; *p* value: 0.281) than the refugee patients when other factors were held constant. Furthermore, the model projected those Ugandan patients were less likely to have strains of lineage 3 (OR = 0.298), both lineages 3 and 4 (OR = 0.868) than the non-Ugandan patients compared to having lineage 4 non-Ugandan family (reference category); however, this was not statistically significant (*p* > 0.05). Conversely, the study showed that Ugandan patients were 1.342 times more likely to have the unclassified strains (95% CI: 0.130-13.54) than the refugee patients when other factors were held constant, but this was not statistically significant (*p* > 0.05).

## 4. Discussion

To gain insight into the MTBC population structure causing PTB in southwestern Uganda, a region where TB infection is widespread, we utilized SNP typing to analyze 283 sputum samples from Ugandan and non-Ugandan patients diagnosed with PTB. SNP typing was chosen since it was optimized and found to be discriminative by Wampande and colleagues [37]. Our findings revealed heterogeneity of MTBC causing PTB in this area, with L4 being the most predominant lineage, with L4-U family and L4-NU accounting for 59.7% and 14.5% of all cases, respectively. The L4-U family was represented by 51.6% of Ugandan and 8.1% of non-Ugandan patients while L4-NU was represented by 13.4% and 1.1%, respectively. Our results of L4 being the most prevalent in this study are in keeping with reports that indicate TB epidemic in Africa is primarily caused by L4 strains [15]. L4 is phylogenetically complex, with at least ten distinct sublineages that differ in geographical distribution, with local genotypes accounting for a larger proportion of circulating strains in certain regions [15]. For instance, the L4-Cameroon family is almost exclusively found in West Africa [14] while the Latin American-Mediterranean (LAM) family is most predominant in Zambia [38, 39]. Similarly, the L4-U family is more predominant in Uganda as opposed to anywhere else [4, 28, 31, 40]. This is supported by our findings which show that L4-U is most common among the Ugandan patients, as opposed to the refugees and comparable with the 63% recorded in central Uganda [28] and 59.2% reported in an earlier study conducted in Mbarara, southwestern Uganda [31]. Studies indicate that in neighboring countries, the L4-U family is less common. Tanzania, for example, reported 21.8% [40] of L4-U, while Kenya reported 11% [41]. It is thus tempting to speculate that local strains are more likely to transmit in a given local setting compared to others.

Another lineage observed in this study at a relatively high proportion was L3, with a prevalence of 15.2%. The Ugandan patients accounted for 12% of this percentage while non-Ugandans accounted for 3.2%. This finding is comparable to the results of a study conducted in central Uganda, which found L3 to be at 11% [28]. Studies conducted in Uganda's neighboring countries such as Tanzania, Sudan, Kenya, and Rwanda have also revealed that L3 (particularly the Central Asian (CAS) family) is a widely implicated lineage in PTB [40]. The MTBC is known to remain stable even in broad cosmopolitan areas such as San Francisco or London where some level of intermingling between locals and immigrants is expected [42]. This is supported by several studies that have shown that MTBC preferably transmits in sympatric host populations [7, 9, 11, 43]. Due to these observations, it is thus easy to speculate that different MTBC lineages could have adapted to different human populations, possibly as a result of MTBC's long coevolutionary history and its human host [6, 8, 17, 18] and that local strains are more likely to transmit in a given local setting compared to others. Our observation that L4-U is predominant in this region is consistent with this hypothesis. However, further work is needed to validate this theory, including studies exploring the interaction between MTBC genetic variation and humans. In our study, 6.4% of the samples were not placed in either of the three lineages screened and may warrant their further characterization to better understand them and to add our knowledge to the molecular epidemiology of TB in this area of Uganda.

## 5. Limitations of the Study

The use of three unique SNP markers in this study may have restricted our ability to detect other MTBC lineages causing PTB in this region. Nonetheless, our research used SNP markers validated by a local study and took into account the common lineages in circulation in east and central Africa, where the patient population is from.

## 6. Conclusion

There is heterogeneity of MTBC causing PTB in southwestern Uganda with lineage 4 Ugandan family strains being the most predominant. However, this diversity needs to be ascertained further with more discriminative techniques such as Mycobacterial Interspersed Repeat Units-Variable Number of Tandem Repeat (MIRU-VNTR) typing or whole genome sequencing.

## Figures and Tables

**Table 1:** Distribution of patient variables across the MTB lineages in southwestern Uganda.

Variable	Category	Patient's characteristics *n* (%)	MTB lineage					*p* value^∗^
			L4-U (*n* = 169)	L4-NU (*n* = 41)	Both L4 and L3 (*n* = 12)	L3 (*n* = 43)	Unclassified (*n* = 18)	
Gender	Male	207 (73.1)	120 (42.4)	34 (12)	9 (3.2)	32 (11.3)	12 (4.2)	0.586
	Female	76 (26.9)	49 (17.3)	7 (2.5)	3 (1.1)	11 (3.9)	6 (2.1)	

Age	18-45 years	209 (73.9)	125 (44.2)	29 (10.2)	9 (3.2)	33 (11.7)	13 (4.6)	0.944
	46-65 years	53 (18.7)	29 (10.2)	9 (3.2)	3 (1.1)	8 (2.8)	4 (1.4)	
	≥66 years	21 (7.4)	15 (5.3)	3 (1.1)	0 (0)	2 (0.8)	1 (0.4)	

HIV status	Positive	76 (26.9)	42 (14.8)	12 (4.2)	4 (1.4)	14 (4.9)	4 (1.4)	0.813
	Negative	78 (27.6)	51 (18.0)	9 (3.2)	2 (0.7)	9 (3.2)	7 (2.5)	
	Unknown	129 (45.6)	76 (26.9)	20 (7.1)	6 (2.1)	20 (7.1)	7 (2.5)	

Nationality	Ugandan	246 (86.9)	146 (51.5)	38 (13.4)	11 (3.9)	34 (12.0)	17 (6.0)	0.319
	Non-Ugandan	37 (13.1)	23 (8.1)	3 (1.1)	1 (0.4)	9 (3.2)	1 (0.4)	

Income status	High	23 (8.1)	13 (4.6)	4 (1.4)	1 (0.4)	4 (1.4)	1 (0.4)	0.980
	Low	260 (91.9)	156 (55.1)	37 (13.1)	11 (3.9)	39 (13.8)	17 (6.0)	

TB in the past	No	266 (94)	156 (55.1)	39 (13.8)	12 (4.2)	41 (14.5)	18 (6.4)	0.503
	Yes	17 (6)	13 (4.6)	2 (0.7)	0 (0)	2 (0.7)	0 (0)	

PTB diagnostic results	High/>5AFB/field	148 (52.3)	82 (29)	19 (6.7)	7 (2.5)	31 (11)	9 (3.2)	0.074
	Low/≤5AFB/field	135 (47.7)	87 (30.7)	22 (7.8)	5 (1.8)	12 (4.2)	9 (3.2)	

^∗^ *p* value obtained by chi-square statistic.

**Table 2:** Multinomial analysis of patient's nationality for MTB lineages in southwestern Uganda.

MTB lineage^a^	Predictors	*B* coefficients	*p* value^∗^	Odds ratio®	95% CI
Lineage 4 Ugandan family	Intercept	2.037	0.001		0.143–1.758
	Ugandan	-0.691	0.281	0.501	
	Non-Ugandan	0	—	—	

Both lineage 4 and lineage 3	Intercept	-1.099	0.341	—	0.082–9.203
	Ugandan	-0.141	0.907	0.868	
	Non-Ugandan	0	—	—	

Lineage 3	Intercept	1.099	0.099	—	0.075–1.193
	Ugandan	-1.210	0.087	0.298	
	Non-Ugandan	0	—	—	

Unclassified	Intercept	-1.099	0.341	—	0.130–13.54
	Ugandan	0.294	0.805	1.342	
	Non-Ugandan	0	—	—	

^a^Reference category is lineage 4 non-Ugandan family; ^∗^*p* value obtained by logistic regression analysis; ®adjusted OR = odds ratio.

## Data Availability

Information used in the study can be accessed at http://www.re3data.org/.

## Abbreviations

CAS: Central Asian family
DNA: Deoxyribonucleic acid
EAI: East-African-Indian
L3: *Mycobacterium tuberculosis* lineage 3
L4: *Mycobacterium tuberculosis* lineage 4
L4-NU: *Mycobacterium tuberculosis* lineage 4 other than Ugandan family
L4-U: *Mycobacterium tuberculosis* Ugandan family
LAM: Latin American-Mediterranean
MTB: *Mycobacterium tuberculosis*
MTBC: *Mycobacterium tuberculosis* complex
PTB: Pulmonary tuberculosis
RT-PCR: Real-time polymerase chain reaction
SNP: Single nucleotide polymorphism
TB: Tuberculosis.

## Supplementary Materials

Supplementary 1 Figure 1: map of Uganda showing the study area.

Supplementary 2 Table 1: lineage-specific SNP typing primers and probes used in the identification of the MTB lineages using RT-PCR assay.

Supplementary 3 Figure 2: an illustration of the RT-PCR (Bio-Rad CFX96 Touch™) SNP-typing amplicon melting temperature analysis using lineage-specific primers and probes.
