# Supplementary figures and images for: Diversity of Mycobacterium tuberculosis Complex Lineages Associated with Pulmonary Tuberculosis in Southwestern, Uganda
Source: Tuberc Res Treat. 2021 Jun 25;2021:5588339. doi: 10.1155/2021/5588339 (PMC8264515; doi:10.1155/2021/5588339)

Figure 1: Map of Uganda showing the study area


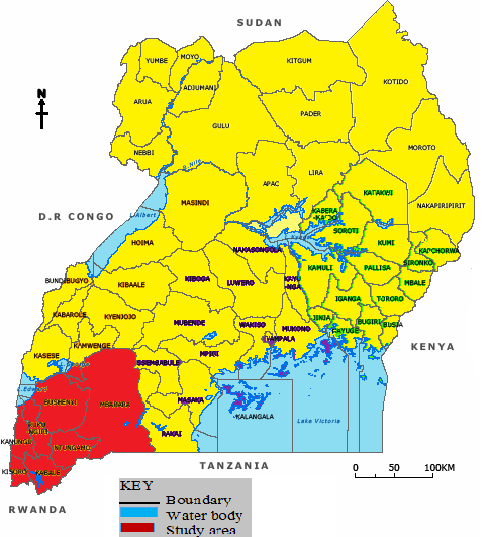

Supplement: Supplementary 1 — Figure 1: map of Uganda showing the study area. [file 5588339.f1.docx]
