# Supplementary material for: Diversity of Mycobacterium tuberculosis Complex Lineages Associated with Pulmonary Tuberculosis in Southwestern, Uganda
Source: Tuberc Res Treat. 2021 Jun 25;2021:5588339. doi: 10.1155/2021/5588339 (PMC8264515; doi:10.1155/2021/5588339)
Supplement: Supplementary 2 — Table 1: lineage-specific SNP typing primers and probes used in the identification of the MTB lineages using RT-PCR assay. [file 5588339.f2.docx]

| **Gene (ORF)** | **SNP position** | **Lineage/ sub-lineage** | **Primers** | **Probes** |
| --- | --- | --- | --- | --- |
| Rv004c | 0619 | MTB Uganda family  (MTB L4-U) | F: 5-ATTGCTCGCATGGCAGA-3  R: 5-AAACCAGGTACTTGTCGG-3 | Probe1: LC Red 640-TGATGACGGAAAGCCGTCGAAA - Pho-3  Probe2: GTTTTCGCGGTAGGTGCCCTCGATG-Fluo-3 |
| Rv2962c | 0711 | MTB lineage 4  (MTB L4-NU) | F: 5-GAACGCCCTTTGCTCTTC-3  R: 5-CAAGGTACTCGTGGTTGG-3 | Probe1: LC Red-CCCGAGCTGATGCCCACCT-Pho-3  Probe2: 5-CACACCCTGTATGCGACG-Fluo-3 |
| Rv0129c | 0472 | MTB Lineage 3  (MTB L3) | F: 5-CGACTGGTATCAGCCCTC-3  R: 5GGAACTGCTGCGGGTAGTA-3 | Probe1: LC Red-GACACGCCTTGTTGGCC-Pho-3  Probe2: 5-CGCCGCGTTGCCTGTCG-Fluo-3 |
