# Supplementary material for: Diversity of Mycobacterium tuberculosis Complex Lineages Associated with Pulmonary Tuberculosis in Southwestern, Uganda
Source: Tuberc Res Treat. 2021 Jun 25;2021:5588339. doi: 10.1155/2021/5588339 (PMC8264515; doi:10.1155/2021/5588339)
Supplement: Supplementary 3 — Figure 2: an illustration of the RT-PCR (Bio-Rad CFX96 Touch™) SNP-typing amplicon melting temperature analysis using lineage-specific primers and probes. [file 5588339.f3.docx]

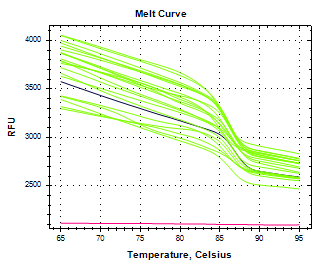

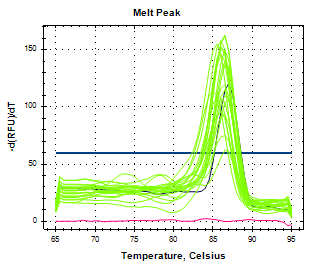


1. b)

Figure 2: An illustration of the RT-PCR (Bio-Rad CFX96 Touch™) SNP-typing amplicon melting temperature analysis using Lineage-specific primers and probes. a) melt temperature of the target region b) derived melting curve. Green = sample; Black = positive control and Red = Negative control.
